# Mental Health Stigma Reduction Interventions Among Men: A Systematic Review

**DOI:** 10.1177/15579883241299353

**Published:** 2024-11-22

**Authors:** Jack Sweeney, Shane O’Donnell, Emilie Roche, P. J. White, Paula Carroll, Noel Richardson

**Affiliations:** 1 South East Technological University, Carlow, Ireland; 2 Institute of Technology Carlow, Carlow, Ireland; 3 South East Technological University, Waterford, Ireland

**Keywords:** systematic review, men, mental health, stigma, stigma reduction intervention, behavior change techniques

## Abstract

Stigma surrounding mental health, particularly among men, remains a significant barrier to men engaging with support services for their mental health. Despite increasing evidence of interventions targeting different aspects of stigma reduction, there is a notable gap in the literature concerning male-specific mental health stigma reduction interventions and on the underlying behavior change techniques (BCTs) used to reduce stigma. The purpose of this review is to synthesize the evidence relating to the impact of mental health stigma reduction interventions among men and to explore the underlying BCTs associated with each intervention. The review was restricted to empirical research reporting on interventions targeting mental health stigma in male-dominated populations. The quality appraisal was conducted using the Mixed Methods Analysis Tool and a narrative synthesis was conducted. Fourteen articles reporting on 11 interventions were included for review, while 20 outcome measures were used. Perceived public stigma attracted the largest number of interventions with a lesser focus on self or personal stigma. Nineteen BCTs were identified across the interventions with information about health consequences and self-monitoring of behavior being the most common followed by credible source, social contact, and behavior practice/rehearsal. This is discussed in relation to the wider literature. The methodological issues highlighted in the articles limit the conclusions and recommendations that can be drawn from the review. Recommendations for further research include standardizing the scales used in stigma measurement, in-depth reporting of intervention descriptions, and greater use of theory to guide intervention development.

## Introduction

Men’s mental health has received increased global media and scholarly attention in recent years, especially in terms of unraveling and addressing the unique challenges and barriers men face in the context of mental health and help-seeking (Lynch et al., 2016; Rasmussen et al., 2018). The attention is, in part, driven by the male suicide rate, which in high-income countries is, on average, three times greater than the female rate (World Health Organization, 2018). Despite this, females have higher reported rates of suicide ideation, self-harm, and suicide attempts (Boyd et al., 2015), as well as higher rates of common mental health problems such as anxiety and depression (Girgus et al., 2017). This “gender paradox in suicide” (Canetto & Sakinofsky, 1998) is not necessarily an indication of better mental health outcomes among men. Rather, it reflects men’s increased likelihood to use more lethal methods for suicide, their lower likelihood to engage with service providers around their mental health, and/or to be formally diagnosed with a mental health problem (Martin et al., 2013; Seidler et al., 2018), leading to underreporting of suicide ideation, self-harming behaviors, and suicide attempts by men. Central to this gender paradox in mental health and suicide behavior is the relationship between mental health stigma, traditional masculine ideologies, and delayed help-seeking (Canetto & Sakinofsky, 1998).

“Stigma” is a multifaceted construct that encompasses people’s knowledge (understanding and awareness of mental health stigma), attitudes (the beliefs and values associated with mental health), and behaviors (the actions and behaviors that perpetuate or challenge stigma) toward individuals, groups, or communities who are perceived to be different or to deviate from the norm (Link & Phelan, 2001; Thornicroft et al., 2009). Stigma can often present as a set of stereotypes, negative emotional responses, prejudicial attitudes, discriminatory behaviors, biased social structures, and/or power asymmetry in some form toward members of a subgroup (Corrigan, 2000). Stigma is therefore present when there is a perception of difference between groups of people, and where these differences are associated with negative or undesirable traits. One such “undesirable trait” that has been reported to carry a significant amount of stigma is mental ill-health.

Within the literature, stigma has been categorized in a number of ways, from individual, structural, social, and internalized stigma to personal, perceived public and self-stigma (Bos et al., 2013; Link & Phelan, 2001) For the purpose of this study, stigma will be categorized in terms of the most well established and widely studied types of stigma in the literature: perceived public stigma; personal stigma; and self-stigma (Corrigan et al., 2014; Link & Phelan, 2001; Pedersen & Paves, 2014). Perceived public stigma relates to the stigmatizing perception about a person who has a mental illness endorsed collectively by members of the general population as they are deemed to have socially undesirable traits (Corrigan et al., 2012; Corrigan & Penn, 1999; Schnyder et al., 2018). Personal stigma therefore describes an individual’s personal attitude toward those deemed to have undesirable traits (Pedersen & Paves, 2014; Schnyder et al., 2018), and self-stigma is where a stigmatized individual may internalize perceived prejudices and develop negative feelings about themselves (Latalova et al., 2014).

Mental health-related stigma can be categorized based on the type of mental illness, such as depression, whether it is experienced or anticipated, and whether it relates to the mental health difficulty itself or seeking help for the mental health difficulty (Corrigan et al., 2003). Mental health stigma has been shown to be a barrier to help and health seeking (Clement et al., 2015; Schomerus et al., 2019), engagement with care (Corrigan, 2004), and adherence to treatment (Abdisa et al., 2020). Further consequences of stigma toward mental ill-health include decreases in employment and social opportunities and the worsening of symptoms for certain mental illnesses such as anxiety or depression (Sickel et al., 2014). Mental health stigma has also been shown to be relationship and context-specific (Major & O’Brien, 2005) and manifests differently in specific social contexts (Dalky, 2012). Therefore, the cultural and social background of the individual can affect their levels of perceived public, personal, and self-stigma.

Stigma is intrinsically linked to masculinity and to different hierarchies within and between masculinities (Mostoller & Mickelson, 2024). The pursuit of more dominant or hegemonic constructions of masculinity (Connell & Messerschmidt, 2005; Malonda-Vidal et al., 2021) pertains to a set of values established by men in power that function to exclude or subordinate others (women and other men) and organize society in gender unequal ways. It represents the idealized or culturally prescribed characteristics, behaviors, and roles that are associated with being a man in a position of power and authority and reflects the interplay between men’s identity, men’s ideals, interactions, power, and patriarchy (Jewkes & Morrell, 2012). The pursuit of hegemonic masculinity can play a role in stigmatizing mental health difficulties (Clark et al., 2020). This is manifested through the suppression/restriction of behaviors such as being vulnerable, crying, or showing fear, in favor of more externalizing “coping” strategies such as substance misuse, risk-taking, and poor impulse control (Oliffe et al., 2019). These strategies align with some of the more traditional masculine ideologies of stoicism, invulnerability, and self-reliance. The paradox or double burden for many men is that this can lead to increasing powerlessness and worsening depression and anxiety (McDermott et al., 2018)—and yet, seeking help cannot be countenanced (Chatmon, 2020), especially if it is perceived to challenge their self-perception of being strong, independent, and self-reliant (Smith et al., 2022). A qualitative review (McKenzie et al., 2022) found that men feel shame, fear, and isolation due to perceived negative attitudes about mental illness in society. The review noted that stigma exists, in particular, in male-dominated environments and that “inequity driven stigma,” relating to issues such as sexual orientation and race/ethnicity, compounds marginalization in male mental health.

A number of previous systematic reviews relating to the stigma of mental health have focused on perceived public, personal, and self-stigma within the general population (Corrigan et al., 2015; Dalky, 2012; Griffiths et al., 2014; McCullock & Scrivano, 2023; Morgan et al., 2018; Pedersen & Paves, 2014; Thornicroft et al., 2016). In the main, these have found evidence for the effectiveness of stigma reduction interventions. These reviews report the use of mechanisms such as education and social contact, that is, contact with people with lived experience (PWLE) and/or contact with the researchers and resources, which are linked with reductions in mental health stigma (Corrigan et al., 2015; Dalky, 2012; Griffiths et al., 2014; McCullock & Scrivano, 2023; Morgan et al., 2018). The most recent review at the time of writing (McCullock & Scrivano, 2023) observed that, in general, interventions targeting self-stigma showed inconsistent results for effectiveness. In their narrative review, Thornicroft et al. (2016) determined that social contact was particularly effective in addressing knowledge of, and behavior toward, mental health in the short term, but that there was limited evidence of long-term effectiveness. Despite these findings, there remains a notable gap in the literature concerning male-specific mental health stigma reduction interventions. The focus of systematic reviews in this area to date has almost exclusively been on stigma reduction among the general population. Existing reviews on male-specific stigma reduction primarily address the stigma surrounding HIV (Dunbar et al., 2020; Heijnders & Meij, 2007; Rosengren et al., 2021). This gap is significant considering the specific and compounding experiences of mental health stigma that men face (McKenzie et al., 2022). Given the fact that stigma reduces help-seeking behaviors (Schnyder et al., 2018), it is imperative that there is a greater understanding of stigma reduction interventions for men.

This review aims to address this gap by assessing the impact of stigma reduction interventions among men and synthesizing the underlying mechanisms of change. A key step in understanding these complexities is identifying the observable and replicable components that bring about change, known as behavior change techniques (BCTs; Michie et al., 2013). While previous research has highlighted gender-responsive approaches to engaging men in health interventions (Galdas et al., 2023; Struik et al., 2019), there is a lack of research on identifying and synthesizing BCTs within stigma reduction interventions. This review focuses on the BCTs employed in existing mental health stigma reduction interventions among men, aiming to shed light on effective strategies for targeting stigmatizing behaviors and attitudes. To the best of the authors’ knowledge, there has been no previous application of this approach to male-specific mental health stigma reduction interventions.

By synthesizing the findings from relevant articles, this review will not only shed light on the present state of stigma reduction interventions targeting men but will guide future research and practice toward evidence-based strategies in this area. Findings will inform more targeted and effective interventions, thereby contributing meaningfully to global conversations on men’s mental health and suicide prevention.

## Method

Articles were restricted to empirical research, written in the English language, and published in peer-reviewed journals. Articles were included if they evaluated the impact of a health intervention on reducing stigma associated with mental health as a primary, secondary, or additional outcome. Articles were not limited by study design. A health intervention was defined as any effort, activity, or combination of program elements designed to improve mental health outcomes (O’Cathain et al., 2019). This review placed a specific focus on adult men but due to the limited number of articles available, a pragmatic approach was taken to include articles if males accounted for a significant majority of ≥70% of participants.

**Figure 1.**
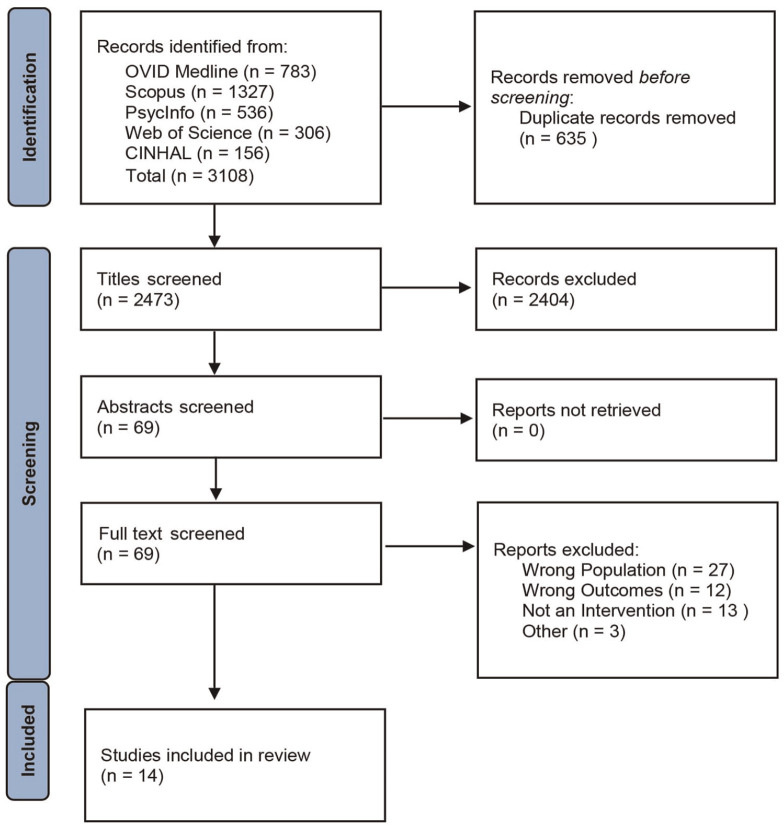
PRISMA Flow Diagram *Note*. PRISMA = Preferred Reporting Items for Systematic Reviews and Meta-Analyses.

This systematic review is reported using Preferred Reporting Items for Systematic reviews and Meta-Analyses (PRISMA) guidelines and was registered on PROSPERO (CRD42022301469). Articles were identified through a systematic search of five electronic databases (Web of Science, OVID Medline, CINHAL, SCOPUS, and PsycINFO) on 22/07/22 and were updated on 12/12/23 with no date limitations. The search string was designed in consultation with a research librarian, informed by previous reviews (Clement et al., 2015; Parcesepe & Cabassa, 2013; Rao et al., 2019), refined through an iterative process, and included a mix of MeSH terms and keywords (See Supplementary File 1). The database search results were imported and organized in Endnote 20. Duplicates were removed, and the remaining references were exported to RAYYAN. Titles were screened against the eligibility criteria independently by two reviewers. This process was repeated with abstracts and finally full texts for included articles in line with the Prisma flow chart (Figure 1). Discrepancies were arbitrated by a third reviewer, although levels of agreement were high (97.1%). Hand-searching of the reference lists of included full texts was conducted to identify relevant papers that may have been missed in the electronic database search.

A standardized data extraction form was developed and pilot-tested to ensure its relevance to the research question. This included study design, setting, participant and intervention characteristics, stigma outcomes, mechanisms of change, and indicators of acceptability and feasibility. Two reviewers independently extracted the data from all the included articles to ensure consistency and reliability within the data extraction process.

## Results

### Study and Intervention Characteristics

A total of 14 articles were included in this review following the screening process (Figure 1), full details of which are shown in Table 1. This comprised 11 quantitative (Fung et al., 2020, 2021; Hanisch et al., 2017; Kohrt et al., 2021; Milner et al., 2018; Nickerson et al., 2020; Sayers et al., 2019; Shimotsu et al., 2014; Syzdek et al., 2014; Tynan et al., 2018; Van Voorhees et al., 2012); two qualitative (Morrow et al., 2020; Robinson et al., 2013); and one mixed-method (Woods et al., 2020) study. Study designs were categorized according to the definitions provided by the Mixed Methods Appraisal Tool (MMAT). In relation to quantitative study designs, six adopted an experimental design—four random control trials (RCTs; Fung et al., 2020, 2021; Milner et al., 2018; Nickerson et al., 2020) and two pilot RCTs (Kohrt et al., 2021; Syzdek et al., 2014), and five were non-randomized studies—four adopted a pre-post test design without control (Hanisch et al., 2017; Shimotsu et al., 2014; Tynan et al., 2018; Van Voorhees et al., 2012) and one a non-randomized trial with a comparison group (Tynan et al., 2018); and there was one time series study (Sayers et al., 2019). One mixed-method study was included which adopted a convergent design (Woods et al., 2020). These studies were conducted in Australia, the United States, the United Kingdom, Canada, Germany, Japan, and Nepal over timeframes ranging from 6 weeks to 4 years. The sample size ranged from 10 to 962 participants, with a pooled total of 4972 participants, of which 91.9% were male.

**Table 1.** Study Characteristics

Author	Country	*n* (% male)	Population	Design	Time	Stigma type	Outcome measure(s)	Intervention description	Results	MMAT
Milner et al. (2018)	Australia	*n* = 478 (100%)	Construction industry workers	RCT	t1=baseline t2=6 weeks	Self-Stigma	SSDS	Six-week brief contact intervention where one message with hyperlinks to information on stigma and mental health literacy was sent via text message per week.	• No significant improvement in SDSS from intervention? vs. control	* * * *
Sayers et al. (2019)	Australia	*n* = 1,651 (83.6%)	Mining industry workers	Repeated cross-sectional survey	t1=baseline; t2=6 months; t3=18 months;	Public stigma	Three items from PSS	Multilevel, peer-based suicide prevention program. Evaluation of combined impact of 1-hour general awareness training; 1-day SafeTalk component and 2-day ASIST training.	• Significant change across all time points in two items relating to attitudes toward mental health (not treated differently by friends or by colleagues due to mental illness; *p* < .01). No significant change to item “not being treated poorly in workplace due to mental illness.”	* * *
Tynan et al. (2018)	Australia	*n* = 1,277 (86.6%)	Mining industry workers	Non-Randomized Trial	t1=baseline t2=post intervention	Public Stigma	One item from PSS	Multilevel, peer-based suicide prevention program. Evaluation of 1-hour general awareness training; 1-day Safe Talk Training; and 2-hour manager training.	• No significant difference within intervention (*p* < .360) or control group (*p* < .284) from T1 to T2 on PSS.	* * *
Nickerson et al. (2020)	Australia	*n* = 103 (100%)	Refugee men with at least one PTSD symptom	RCT	t1=baseline t2=post intervention t3=4 weeks follow-up	Self-Stigma	SSDS (adapted for PTSD); SSSH	Four-week program with 11 interactive web-based modules to reduce stigma and increase help-seeking using psychoeducation (PE), social contact, and cognitive reappraisal of negative beliefs about mental health and help-seeking.	• No significant effect on SSDS (adapted for PTSD). • Significant improvement in SSSH from T2 to T3 for intervention compared with control (*p* < .022).	* * *
Fung et al. (2021)	Canada	Total *n* = 495 (100%) ACT *n* = 133 (100%) CEE *n* = 149 (100%) ACT and CEE *n* = 152 (100%)	Asian men with experience living with or affected by a mental health issue.	RCT	t1=baseline t2=post intervention t3=3 months post t4=6 months post	Public and Self-Stigma	CAMI, ISMI, SJS	Anti-stigma interventions for Asian immigrant men in Canada in the community setting using Acceptance and Commitment Training (ACT), Contact-based Empowerment Education (CEE), and PE focusing on storytelling, dialogue, capacity building, and identifying challenges and opportunities for stigma reduction.	**PE** • No effect on any subscales of CAMI. • No effect on any subscales of ISMI. **ACT** • Empowerment mediated a significant effect on CAMI, psychological inflexibility, alienation, and resistance to mental illness stigma on ISMI. **CEE** • Empowerment mediated a significant effect on all CAMI subscales. • Mindfulness mediated a significant effect on benevolence. • Empowerment mediated a significant effect on stigma resistance. • Mindfulness mediated an effect on social withdrawal.	*
Fung et al. (2020)	Canada	Total *n* = 535 (100%) ACT *n* = 145 (100%) CEE *n* = 160 (100%) ACT & CEE *n* = 164 (100%) PE *n* = 66 (100%)	Asian men with experience living with or affected by mental health.	RCT	t1=baseline t2=post intervention t3= 3 months post t4= 6 months post	Public and Self-Stigma	CAMI, ISMI, SJS	Anti-stigma interventions for Asian immigrant men in Canada in the community setting using ACT. CEE, and PE focusing on storytelling, dialogue, capacity building, and identifying challenges and opportunities for stigma reduction.	**PE** • Significant effect on authoritarianism and social restrictiveness. No effect on other CAMI subscales. • No effect on any subscales on ISMI. **ACT** • Significant effect on Authoritarianism. • No other effect on other CAMI subscales. • Significant effect on self-stigma, particularly on alienation, stereotype endorsement, social withdrawal, and stigma resistance but no effect on discrimination experience. Significant effect on behavior control and behavior intention subscale of SJS. **CEE** • Significant effect on authoritarianism and social restrictiveness. • No effect on other CAMI subscale. • Significant effect on self-stigma overall, in particular, alienation and stigma resistance. • No significant effect on stereotype endorsement, discrimination experience, or social withdrawal. Significant effect on behavioral control and subjective norms subscale of SJS.	*
Morrow et al. (2020)	Canada	*n* = 94 (100%)	Asian men with experience living with or affected by a mental health issue.	Qualitative Focus Groups	t1=baseline t2=post intervention			Anti-stigma interventions for Asian immigrant men in Canada in the community setting using ACT. CEE, and PE focusing on storytelling, dialogue, capacity building, and identifying challenges and opportunities for stigma reduction.	• Increased awareness of masculine ideals and greater ability to disrupt hegemonic masculine norms. • Increase in willingness to assist those in need of help, especially those of their own ethnicity.	* * * *
Robinson et al. (2013)	United Kingdom	*n* = 9 (77.7%)	Members of the general public	Qualitative	t1=Post intervention	Public Stigma		Six-year public awareness raising campaign, primarily targeting male-specific settings, to raise awareness of crisis service numbers, challenge stigma around suicide, and encourage help-seeking.	• Increased awareness of suicide and crisis service numbers which was attributed to the routine of messages in a trusted setting. • Increase in openness to talk about vulnerability, feeling low, or suicidal thoughts with reports of increased intention to seek help. • Community settings appealed to target groups and more widespread appeal was important.	* *
Woods et al. (2020)	United Kingdom	Survey *n* = 75 (100%) Focus Group *n* = 15 (100%)	British Cat C male prisoners	Mixed Methods with a convergent design	t1=baseline t2=post intervention t3= 8 weeks post	Personal Stigma, Knowledge relating to stigma	MAKS, RIBS	A single 75-minute face-to-face program aimed at raising awareness of and promoting psychological well-being and resilience, tackling stigma, and highlighting the importance of signposting to appropriate services through the presentation of two case studies from former elite rugby players and the presentation of risk factors, markers of stress, and coping strategies.	• Significant improvements in mental health knowledge • No significant effect on mental well-being • No significant effect on resilience	* *
Syzdek et al. (2014)	America	*n* = 23 (100%)	Community-dwelling men who screened positive for anxiety and depression with no history of formal help-seeking	Pilot RCT	t1=baseline t2=1 month post t3=3 months post	Self-Stigma	PPL	A 2-hour gender-based motivational interview via a computer program which included a 30-minute intake interview, a 30-minute computerized assessment, and a 60-minute feedback interview.	Reported a small effect size on self-stigma, but was attributed to an increase in self-stigma among the control group also.	* *
Van Voorhees et al. (2012)	America	*n* = 50 (90%)	Veterans with combat-related mental distress	One-Group before and after the study	t1=baseline t2=4 weeks post t3=8 weeks post t4=12 weeks post	Self-Stigma	SN-TPB	An online CBT and Peer Support Program which consisted of six half-hour sessions.	Significant reduction in both “embarrassment if friends knew I was receiving help” and belief that “others would be disappointed if they had PTSD/Depression,” but no significant change in “I would not want my employer to know that I am receiving help for PTSD/depression.”	* *
Shimotsu et al. (2014)	Japan	*n* = 46 (71%)	Psychiatric outpatients with anxiety and depressive symptoms	One-Group before and after the study	t1=baseline t2=post	Public Stigma	DDS	Ten 60-minute group CBT sessions with the first and last sessions being individual.	Significant Improvements in the DDS scale from pre to post. Changes were strongly correlated with significant changes in depression and Anxiety scores and moderately correlated with dysfunctional attitudes. Self-stigma also acted as a mediator between dysfunctional attitudes and symptoms of anxiety and depression.	* *
Kohrt et al. (2021)	Nepal	*n* = 88 (85%)	Primary Care Practitioners (PCPs) in Chitwan, Nepal	Pilot RCT	t1=baseline t2=4 months post t3=16 months post	Personal Stigma	SDS, mhGAP, IAT	12 sessions totaling 40 hours of training in mhGAP which is a guide to co-facilitate introductions to mental illness, recovery stories, Q&A sessions, and a number of structured and unstructured activities with PWLE and other inspirational figures in the community.	Reduction of 7.8 points on the social distance scale in the intervention compared with the control group; improvements in mhGAP knowledge and attitudes in both the intervention and control group but no within-group improvements in the IAT.	* * * *
Hanisch et al. (2017)	Germany	*n* = 48 (92%)	Managers of multination organizations	One-Group before and after the study	t1=baseline t2=post t3=12 weeks post	Personal Stigma, Knowledge related to stigma	MAK, OMS-WA	1–2 hour single online session where managers ran through a virtual 7 weeks as a manager tasked with supervising a virtual team and managing their mental health effectively, each team member showed diverse psychological profiles and represented different mental health scenarios which were likely to appear in real life.	Significant improvements in stigma-related knowledge from pre to post intervention which was maintained at the 3-month follow-up. Significant decrease in stigmatizing attitudes which was sustained at the 3-month follow-up also. In particular, there were decreases in avoidance, perceived dangerousness, and responsibility but not in work and competency beliefs or helping people with mental illness.	* *

*Note.* SSDS = Self-Stigma of Depression Scale; PSS=Perceived Stigma Scale; SSSH = Self-Stigma of Seeking Help Scale; CAMI = The Community attitudes toward the mentally ill; ISMI = Internalized Stigma of mental Illness; SJS = Social Justice Scale; MAKS = Mental Health Knowledge Schedule; RIBS = Reported and Intended Behavior Scale; PPL = The Perceptions of Problems in Living Questionnaire; SN-TPB = Social norm questions related to the Theory of Planned Behavior; DDS = Devaluation-Discrimination Scale; SDS = Social Distance Scale; mhGAP = the WHO Mental Health Gap action program attitudes assessment; IAT = Implicit Association Test; OMS-WA = Opening Minds Scale for Workplace Attitudes; PWLE = People with lived experience. QAS Scale is based on the MMAT quality assessment tool 2018 (W. N. Hong et al., 2018) and scored accordingly. Each “*” represents adequately passing the one of the five assessment questions.

Fourteen articles reported on 11 distinct interventions. Two interventions were concerned with male ethnic minorities—one online psychoeducational intervention among refugee men with post-traumatic stress disorder (PTSD; Nickerson et al., 2020) and one multicomponent intervention evaluating the impact of acceptance commitment therapy, contact-based empowerment education (CEE), psychoeducation (PE) and a combination of these modalities among Asian Canadian men (Fung et al., 2020, 2021; Morrow et al., 2020). The other two interventions were concerned with gender-based motivational interviewing among men with internalized symptoms of mental health and with no history of help-seeking (Syzdek et al., 2014), and a male-focused, suicide prevention public awareness campaign (Robinson et al., 2013). Three interventions related to reducing mental health stigma in the workplace setting. One related to a peer-based suicide prevention program in the mining industry (Sayers et al., 2019; Tynan et al., 2018); one was a brief contact intervention that sent text messages with hyperlinks to information on mental health and stigma reduction to construction industry workers (Milner et al., 2018); and one related to a leadership mental health promotion training for managers using a simulated game (Hanisch et al., 2017). Two interventions related to reducing stigma in the health care setting—one among primary care practitioners trained in the WHO Mental Health Gap Action Program and co-facilitated by PWLE of mental illness (Kohrt et al., 2021); and one group cognitive-behavioral therapy (CBT) program among psychiatric outpatients with anxiety and depressive symptoms (Shimotsu et al., 2014). Finally, one intervention was concerned with reducing stigma among male prisoners via a sports-centric mental health awareness program (Woods et al., 2020), while another related to an online PE program for veterans with combat-related distress (Van Voorhees et al., 2012). The intervention duration ranged from 75 minutes to 10 weeks—the public awareness campaign ran for 6 years. Nine interventions were face-to-face (Fung et al., 2020, 2021; Kohrt et al., 2021; Morrow et al., 2020; Sayers et al., 2019; Shimotsu et al., 2014; Syzdek et al., 2014; Tynan et al., 2018; Van Voorhees et al., 2012; Woods et al., 2020); three were conducted online (Hanisch et al., 2017; Milner et al., 2018; Nickerson et al., 2020), and one was a public awareness campaign (Robinson et al., 2013). Five interventions focused exclusively on males.

See Table 1 for more details on study and intervention characteristics.

### Quality Appraisal

The MMAT was used to appraise the quality of each paper, each of which was then scored accordingly (W. N. Hong et al., 2018). The MMAT was chosen due to the mixed methods nature of this systematic review (W. N. Hong et al., 2018). This tool has been validated and is widely used in systematic reviews including both qualitative and quantitative-based studies (Q. N. Hong et al., 2019). Quality appraisal was conducted independently by two reviewers (JS and SOD), and disagreements were arbitrated by a third reviewer (PJW).

Across the four methodologies that were identified, the most common flaws in the methodology of the quantitative randomized papers were as follows: (a) did not report on participants’ adherence to the intervention (Fung et al., 2020, 2021; Kohrt et al., 2021; Milner et al., 2018; Nickerson et al., 2020); (b) did not report on assessors being blinded to the intervention (Fung et al., 2020, 2021; Nickerson et al., 2020; Syzdek et al., 2014); and (c) did not have comparable groups at baseline nor appropriate randomization reported or performed (Fung et al., 2020, 2021; Syzdek et al., 2014). The most common flaws in the methodology of the quantitative non-randomized papers were as follows: (a) confounders were not reported or accounted for and (b) noncomplete outcome data. The most common flaws in the methodology of the qualitative papers were as follows: (a) findings were not adequately derived from the data. The most common flaws in the methodology of the mixed methods papers were as follows: (a) lack of a rationale for using mixed methods; (b) different components of the study were not effectively integrated when answering the research question; and (c) outputs of the integration of both components were not adequately interpreted.

The total results are shown in Supplementary File 2.

### Narrative Synthesis

There were 20 separate outcome measures used to assess intervention effects on reducing stigma among the articles in this review. Only two measures were used in more than one intervention—the Self-Stigma of Depression Scale (SDS) and the Mental Health and Knowledge Schedule (MAKS). All outcome measures are outlined in Table 1. The outcomes are discussed under the broad categories of Stigma Knowledge, Stigma Attitudes, and Stigma Behaviors and further delineated between self-stigma, personal stigma, and public stigma where possible.

Two interventions reported significant improvements in stigma-related knowledge using the MAKS. Significant improvements in stigma-related knowledge, 2.16 (0.335), *p* < .001, were reported among German managers post intervention which was also maintained at the 3-month follow-up, 1.87 (0.361), *p* < .001, (Hanisch et al., 2017)—neither age nor education had significant effects on initial status or goodness of fit. While significant improvements were also reported among British prisoners on stigma-related knowledge compared with a control group (5.24, *p* = .03), the control group had lower MAK scores at baseline which may have affected the results (Woods et al., 2020).

### Attitudes

#### Public Stigma

Two interventions reported on public stigma using the perceived public stigma scale (Sayers et al., 2019; Tynan et al., 2018) and qualitative inquiry (Robinson et al., 2013). The peer-based suicide prevention intervention reported a significant change to Australian coal miner’s belief that an employee would not be treated differently by friends or colleagues for having a mental illness (*p* <.01; Sayers et al., 2019). Despite this, there was no significant change to Australian men’s belief that a person would not be “treated poorly” in the workplace more generally (Sayers et al., 2019; Tynan et al., 2018). There was a perception of increased awareness of suicide and crisis service numbers in the general public in the United Kingdom, particularly in areas where campaign resources were concentrated following the public awareness campaign (Robinson et al., 2013). Routinely endorsing messages over time, within trusted settings, and with consistent and innovative messages were reported to be key factors in the campaign’s success.

#### Personal Stigma

Three interventions were reported on personal stigma using four different scales. Overall, there was a general trend toward a reduction in personal stigma. Hanisch et al. (2017) reported a significant decrease in overall personal stigma in German managers using the Attitudes toward coworkers who may have a mental illness (OMS-WA) which was sustained at 3 months following a gamified and simulated PE leadership training program. More specifically, there was a significant decrease in the subscales avoidance (–1.66 [0.422], *p* < .001), responsibility (–0.68 [0.225], *p* = .003), and perceived dangerousness (–1.51 [0.333], *p* < .001), but attitudes relating to work and competency beliefs, and helping people with a mental illness did not change. Fung et al. (2021) reported a significant decrease among Asian Canadians in perceived threat to society (*t* = 3.78; confidence interval [CI], 0.74–2.34), in the Community attitudes toward the mentally ill (CAMI) scale following PE, CEE, and/or a combination of acceptance commitment therapy (ACT) and CEE, while all four intervention arms also led to a significant decrease in the belief that people with mental illness are inferior and need custodial care. Only CEE significantly increased compassion and goodwill toward people with mental illness and community ideology approach to reducing stigma (*t* = 3.68; CI, 0.68–2.25). Further analysis reported that empowerment had a significant mediating effect on all subscales following all intervention arms (Fung et al., 2020). Finally, there was a within-group improvement in Mental Health Gap Action Program (mhGAP) attitudes and knowledge scores (19.2 [16.0–22.4]) following a co-produced version of mhGAP Intervention Guide (mhGAP-IG) among primary care practitioners, but these improvements for Nepalese primary care practitioners were observed in both the intervention and control groups (Kohrt et al., 2021). Indeed, there was a wide variation in study design, quality, and outcome measures, which means that these results should be interpreted with caution.

#### Self-Stigma

Five interventions were reported on self-stigma attitudes using six different outcome measures. Four interventions used validated scales—the Self-Stigma for Depression Scale (Milner et al., 2018) which was also adapted for PTSD (Nickerson et al., 2020); the Internalized Stigma of Mental Illness Scale (Fung et al., 2020, 2021); and the Devaluation-Discrimination Scale (Shimotsu et al., 2014)—while the remaining two articles used ad hoc questions (Van Voorhees et al., 2012) and a measure with no reported psychometric properties (Syzdek et al., 2014). Overall, there appears to be little to no evidence that PE, empowerment, or contact with a person with mental illness reduces self-stigma. Neither of the RCTs reported a significant effect on Australian construction workers’ self-stigma of depression or Asylum-seeking/refugees’ in Australian’s self-stigma of PTSD following the completion of SMS and/or online interventions which largely focused on PE and contact with someone with a mental illness (Milner et al., 2018; Nickerson et al., 2020). The intervention arms of PE and CEE in the Fung et al. (2021) study had no significant effect on overall self-stigma for Australian construction workers. While the gender-based motivational interviewing intervention reported a small to moderate effect on American men with anxiety, this may be because of a greater increase in self-stigma among the control group post intervention and at follow-up rather than a decrease among the intervention group (Syzdek et al., 2014).

There is some evidence that the interventions underpinned by a psychotherapeutic approach can reduce self-stigma. A 10-week CBT program by Shimotsu et al. (2014), significantly reduced self-stigma among Japanese psychiatric outpatients post intervention, *t*(45) = 3.25, *p* < .001. An online CBT and peer support intervention by Van Voorhees et al. (2012), reported a significant increase in American combat veterans acceptance of a mental illness “If my doctor told me that I had PTSD or depression, I could accept that” (*p* < .001), and a reduction in beliefs that they would be embarrassed if a friend knew they were getting professional help for PTSD and/or depression (ES = 0.80, 95% CI = 1.20–0.38). There was no change to the belief that they would not want an employer to know if they were getting professional help for PTSD and/or depression. Fung et al. (2021) also reported a significant improvement in Australian construction workers’ overall self-stigma following engagement with two intervention arms—ACT or a combination of ACT and CEE—but no overall effect for CEE alone or for PE. More specifically, following ACT, there were significant improvements in the subscales stereotype endorsement, social withdrawal, alienation, and stigma resistance, while improvements were only significant for the latter two constructs following ACT and CEE combined. Further analysis highlighted that decreased psychological inflexibility mediated the decrease in alienation while greater empowerment mediated the increase in stigma resistance (Fung et al., 2021).

#### Stigma Behavior

Four interventions were reported on behavioral outcomes of stigma using three scales. The Social Justice Scale was used to measure behavioral outcomes in two articles (Fung et al., 2020, 2021) and reported a significant effect on behavioral control of Australian construction workers from ACT, CEE, and a combination of both. Woods et al. (2020) found no significant effect regarding behavioral stigma for British prisoners on the Reported and Intended Behavior Scale (RIBS) among prisoners in either the intervention or control groups. There was a greater mean reduction of 7.8 points for Nepalese primary care practitioners on the Social Distance Scale (SDS) in the intervention versus the control group in the pilot RCT among health care practitioners (−10.5 [−14.1 to −6.99]) (Kohrt et al., 2021). Finally, following the public awareness campaign, Asian Canadians and participants from the United Kingdom who had not personally experienced mental health challenges reported being more open to talking about mental health and suicide (Morrow et al., 2020; Robinson et al., 2013), to actively reflect and discuss masculinity (Morrow et al., 2020), to challenge stigmatizing attitudes (Morrow et al., 2020; Robinson et al., 2013), to seek help (Robinson et al., 2013), and to have an increased motivation toward assisting others (Morrow et al., 2020).

### BCT Synthesis

This review found that the most utilized BCTs aimed at reducing mental health stigma across the interventions included “information about health consequences” (*n* = 10)—educating participants on the negative impacts of stigma such as isolation and discrimination; “self-monitoring of behavior” (*n* = 10)—facilitating awareness of maladaptive patterns through cognitive-behavioral strategies; “feedback on behavior” (*n* = 7)—providing insights to the effect that isolating behaviors can have on those with mental health challenges; “credible source” (*n* = 6)—the impact of influential public figures or individuals with lived experience conveying information; and “behavior practice/rehearsal” (*n* = 5)—reinforcing supportive actions through positive feedback and repetition.

Moderately common BCTs were “information about social/environmental consequences” (*n* = 4)—illuminating how both negative and positive attitudes affect those with mental illness; “instructions on how to perform behaviors” (*n* = 4)—outlining skills for engaging those with mental ill-health; and “information about antecedents” (*n* = 3)—examining precursors to stigmatizing emotions/conduct.

Less frequent BCTs included “social support” (*n* = 2)—community support systems; “problem solving” (*n* = 2)—developing solutions to stigmatizing attitudes; and “framing/reframing” (*n* = 2)—taking stock of how people with mental ill-health are viewed. Single interventions employed “monitoring emotional consequences”; “feedback on behavior outcomes”; “re-attribution”; and “verbal persuasion about capacity building.”

All study results can be found in Table 2.

**Table 2.** Intervention Function and BCT Summary

Articles	Intervention	BCTs	Examples
Sayers et al. (2019), Tynan et al. (2018)	Multilevel, Peer-Based Suicide Prevention Program (i) GAT training (ii) Connector training (iii) Assist training	4.1. Instructions on how to perform behavior, 3.3. Social Support, 5.1. Information about health consequences, 5.3. Information about social and environmental consequences, 5.6. Information about emotional consequences, 1.4. Action planning, 15.1 Verbal persuasion about capability, 13.2 Framing/Reframing	(i) Increasing mental health and suicide awareness in the given industry through increasing mental health literacy, reducing stigma, and encouraging help-seeking and help-offering. (ii) Increasing help-offering through education about warning signs, how to ask about the topic of suicide and mental health, how to refer to help, and how to look after oneself. (iii) Increasing the ability to help through education about warning signs, how to ask about the topic of suicide and mental health, how to refer to help, and how to look after oneself.
Morrow et al. (2020), Fung et al. (2020, 2021)	(i) Acceptance and commitment training (ACT) (ii) Contact-based empowerment education (CEE), (iii) Both ACT and CEE, (iv) Psychoeducation	2.3. Self-monitoring of behavior, 2.2. Feedback on behavior, 8.1. Behavior practice/rehearsal, 1.7. Reviewing outcome goals,5.1. Information about health consequences, 5.3. Information about social environmental consequences, 4.2 Information about antecedents, 5.6. Information about emotional consequences	(i) Developing skills in mindfulness and psychological flexibility through interactive group exercises. (ii) Education taught through the PWLE representatives going through storytelling, dialogue, and capacity building around stigma and mental health. (iii) Developing skills in mindfulness through interactive group exercises followed by education taught through the PWLE representatives going through storytelling, dialogue, and capacity building around stigma and mental health. (iv) Education taught through storytelling, dialogue, and capacity building around stigma and mental health.
Hanisch et al. (2017)	Leadership training in mental health promotion using a web-based gamified training program.	4.1. Instructions on how to perform behavior, 2.2. Feedback on behavior, 2.7. Feedback on outcomes of behaviors, 8.1. Behavior practice/rehearsal, 2.3. Self-monitoring of behaviors	Participants are run through a virtual 7-day week tasked with the supervision of a virtual team. The participants must manage the team’s mental health effectively, with each member showing diverse profiles with different mental health scenarios which are representative of real life.
Kohrt et al. (2021)	Reducing Stigma among healthcare providers (RESHAPE) intervention	4.1. Instructions on how to perform behavior, 8.1. Behavior practice/rehearsal, 5.1. Information about health consequences, 5.3. Information about social and environmental consequences, 9.1 Credible source, 2.2. Feedback on behavior	Integration of the WHO mhGAP-IG training delivered by PWLE.
Milner et al. (2018)	Contact+Connect Brief contact intervention	5.1. Information about health consequences, 2.3. Self-monitoring of behaviors, 9.1 Credible source, 7.1. Prompts/cues	Providing information on the risk/protective factors for depression and suicide, the importance of social support, communication and help-seeking, and dispelling myths about mental health problems through SMS text messages.
Nickerson et al. (2020)	An online psychoeducation, cognitive reappraisal, and social contact intervention	5.1. Information about health consequences, 2.3. Self-monitoring of behaviors, 9.1 Credible source 4.3 Re-attribution	Online web-based modules which include psychoeducation, cognitive reappraisal, and a social contact aspect.
Robinson et al. (2013)	Public awareness campaign	5.1. Information about health consequences, 4.1. Instructions on how to perform behavior, 3.3. Social support, 1.4 Action planning	The use of publicly accessible media and posters to inform and spread information related to mental health and stigma.
Shimotsu et al. (2014)	Group cognitive-behavioral therapy	2.3. Self-monitoring of behaviors, 13.2 Framing/reframing, 4.3 Re-attribution, 2.2. Feedback on behavior	The use of CBT to reframe the self-stigma and its relationship with cognitive restructuring.
Syzdek et al. (2014)	Gender-based motivational interviewing	2.2. Feedback on behavior, 1.4 Action planning, 5.4. Monitoring of emotional consequences	The use of Gender-based motivational interviewing with a 30-minute computerized assessment and a further 60-minute feedback interview based on the computerized assessment.
Van Vorheer et al. (2012)	Online “Vets Prevail” behavior change intervention	2.3. Self-monitoring of behaviors, 2.2. Feedback on behavior, 1.2 Problem solving, 13.2 Framing/Reframing	The combination of problem-solving therapy, CBT, motivational frameworks, and tailored culturally appropriate materials.
Woods et al. (2020)	Community-based mental health initiative “State of Mind Sport (SOMS)”	5.1. Information about health consequences, 9.1 Credible source, 7.1. Prompts/cues, 2.3. Self-monitoring of behaviors, 13.2 Framing/reframing	An education program delivered through substances misuse nurse and two elite-level rugby players who gave their own personal case studies along with specific contextual information relevant to the prison environment.

## Discussion

The aim of this review was to explore the impact of mental health stigma reduction interventions among men and to synthesize the BCTs used within the interventions. Findings suggest that, in general, interventions were effective in reducing stigma. In particular, PE and social contact were found to be successful in shifting attitudes toward perceived public stigma and personal stigma, whereas a combination of PE and psychotherapeutic approaches improved self-stigma. A synthesis of the BCTs identified clusters of natural consequences (e.g., information about health consequences), and feedback and motivation (e.g., feedback on behavior). However, quality assessment using the MMAT determined the overall quality of the studies to be substandard, with significant omissions in the reporting of critical details.

PE in the form of information about health consequences and social/environmental consequences, which incorporated social contact through delivery by credible sources or PWLE, were common domains of BCTs utilized to target public and personal stigma. This reflects the evidence found in general populations in reducing stigma around mental health (Dalky, 2012; Gronholm et al., 2017; Thornicroft et al., 2016). Regarding PE, it has been shown that possessing accurate knowledge empowers individuals to think critically and counter societal biases instead of adopting more negative perceptions (Yanos et al., 2015). It is important to acknowledge that educational approaches can differ significantly in the content they deliver, and these differences were not reported in the articles under review. Perceived work competency and workplace treatment showed no improvement using PE (Hanisch et al., 2017). In male-dominated workplaces, it may be important to incorporate PE on the cyclical, reinforcing relationship between restrictive masculine pressures like emotional suppression, worsened mental health outcomes, and heightened self-stigma in needing support (Himmelstein & Sanchez, 2014). Regarding social contact, within general populations, direct contact has been found to reduce desired social distance and belief in stereotypes by humanizing those affected (Alexander & Link, 2003). Uncertainties around the specific mechanisms of social contacts’ stigma-reducing effects and its long-term impacts remain unclear (Gronholm et al., 2017; Jorm, 2020). While the quality and conditions of social contact are an important consideration (Gronholm et al., 2017), this could not be established in the reviewed articles. More detailed reporting of the quality and conditions of the contact used in future intervention studies would help determine the specific facilitating conditions that lead to the most effective contact-based interventions for reducing mental health-related stigma. This, in turn, would aid/enable clearer comparisons across studies, lay a framework for others to employ similar facilitation conditions, and foster a more cohesive body of evidence. Prioritizing contact-based destigmatizing dialogue through credible sources and endorsing more flexible masculinities that promote well-being over rigid norms around toughness (Robertson et al., 2018) may be most effective.

Alongside PE, a psychotherapeutic approach that uses elements of ACT and CBT showed benefits for bringing about change to self-stigma (Fung et al., 2020, 2021; Van Voorhees et al., 2012). These therapeutic modalities were found to be important in a previous review of self-stigma reduction strategies in the broader population (Mittal et al., 2012). The rationale behind their effectiveness lies in their ability to address the underlying self-constructs that contribute to stigmatizing oneself by empowering individuals to reframe and challenge ingrained beliefs and thought patterns related to self-stigma. For example, McKenzie et al. (2022) suggest that by reframing mental health issues as physical health concerns or presenting them as common aspects of daily life, some men were able to reduce the stigma surrounding the condition and seek potential treatments. In addition, Syzdek et al. (2014) reported positive effects of incorporating motivational interviewing and “feedback to address self-stigma.” These combined strategies make intuitive sense given that motivational interviewing can enhance an individual’s intrinsic motivation to change, while targeted feedback offers a reflective space for individuals to confront and adjust self-stigmatizing beliefs.

Improvements in stigma behavior, evidenced in this review in a single intervention, used a combination of PE, social contact, cognitive restructuring, and ACT (Fung et al., 2020, 2021). Previous research has demonstrated that contact-based education and ACT are equally effective at increasing empathy toward mental illness, as demonstrated within a nursing student population (Vaghee et al., 2017).

An increase in stigma-related knowledge was reported following training with managers (Hanisch et al., 2017) and PE methods with credible sources targeting prisoners (Woods et al., 2020). The importance of increasing knowledge is heightened by previous research findings showing that a stronger alignment with traditional masculine norms is inversely related to health literacy (Milner et al., 2018). Specifically, Milner (2018) suggests that attributes such as self-reliance, inherent in certain masculine norms, may conflict with communicative and interactive health literacy. As such, the design of interventions targeting stigma-related knowledge among men may have to target the underlying masculine norms prevalent within this group.

The synthesis of BCTs, drawn from the interventions and outcomes detailed above, reveals that a combination of BCTs may provide the most effective approach to stigma reduction across the domains. This includes information about health consequences, for example, educating participants on the negative impacts of stigma. Feedback and monitoring of behavior are highlighted as fundamental components of psychotherapeutic approaches. The integration of credible sources, which in this context refers to credible male role models, can be used to challenge traditional masculine norms and to demonstrate that mental health awareness is congruent with masculine identities. This approach not only educates but also restructures perceptions, making it an essential strategy in engaging men and reducing mental health stigma.

## Limitations and Future Directions

Included papers were limited to those published in the English language, which were peer-reviewed and excluded gray literature. Therefore, it is possible that papers outside these criteria may have been missed. The review identified several methodological issues across the articles that impacted the reliability of the findings. Quantitative research limitations related to participant adherence, blinding, and randomization. Non-randomized studies frequently overlooked potential confounding factors and exhibited data gaps. Qualitative research in some instances lacked robust, data-driven conclusions, and mixed methods studies were lacking in methodological coherence and integration. These issues underscore the need for more rigorous research designs, clear reporting, and better integration of methodologies to ensure reliable outcomes. These are issues that have been raised previously (McCullock & Scrivano, 2023). In addition, the review noted considerable variability in the measures used across articles, with higher-quality research generally showing no significant intervention effects compared with lower-quality studies. This variability, along with differences in socio-cultural contexts and participant backgrounds, made it difficult to directly compare the impact of interventions. It is imperative that future studies address these methodological shortcomings when designing and evaluating mental health stigma reduction interventions. Future research into the mechanisms of change is needed. Delving deeper into the mechanisms through which BCTs exert their effects on stigma reduction and better understanding the psychological processes underlying behavior change can inform the development of more targeted and efficient interventions.

## Conclusion

This systematic review provides much-needed insights into the impact of existing mental health stigma reduction interventions among men. The review addresses a notable gap in the literature as the first review to specifically synthesize evidence on interventions tackling male mental health stigma through the lens of the BCW, specifically BCTs. Findings demonstrate improvements across interventions in perceived public stigma, personal stigma, and self-stigma. PE and social contact consistently emerged as effective approaches for shifting attitudes, while self-stigma decreased through combined education and therapeutic techniques enabling thought pattern reframing. In addition, there is a need to explicitly challenge more unhelpful masculine norms and stereotypes that may be prevalent within a group when designing destigmatizing content. Credible male role models speaking out and fostering open dialogue have a crucial role to play in normalizing the topic of male mental health.

## Supplemental Material

sj-docx-1-jmh-10.1177_15579883241299353 – Supplemental material for Mental Health Stigma Reduction Interventions Among Men Supplemental material, sj-docx-1-jmh-10.1177_15579883241299353 for Mental Health Stigma Reduction Interventions Among Men by Jack Sweeney, Shane O’Donnell, Emilie Roche, P. J. White, Paula Carroll and Noel Richardson in American Journal of Men's Health

sj-pdf-2-jmh-10.1177_15579883241299353 – Supplemental material for Mental Health Stigma Reduction Interventions Among Men Supplemental material, sj-pdf-2-jmh-10.1177_15579883241299353 for Mental Health Stigma Reduction Interventions Among Men by Jack Sweeney, Shane O’Donnell, Emilie Roche, P. J. White, Paula Carroll and Noel Richardson in American Journal of Men's Health
