# Supplementary material for: Mental Health Stigma Reduction Interventions Among Men: A Systematic Review
Source: Am J Mens Health. 2024 Nov 22;18(6):15579883241299353. doi: 10.1177/15579883241299353 (PMC11585030; doi:10.1177/15579883241299353)
Supplement: sj-docx-1-jmh-10.1177_15579883241299353 – Supplemental material for Mental Health Stigma Reduction Interventions Among Men [file sj-docx-1-jmh-10.1177_15579883241299353.docx]

Five databases (Web of Science, OVID Medline, CINHAL, SCOPUS and PsycINFO) were searched for potentially relevant abstracts published prior to December 2023. The search was undertaken at two time points: July 2022, and December 2023.

The search terms for the study were developed by conducting a preliminary search of Scopus using the terms ABS( stigma* OR “Social Acceptance” OR Attitud*) AND ABS( "mental health"  OR  "mental wellbeing"  OR  "Well-being"  OR  "Mental Illness"  OR  suicid*  OR  "mental Disorder*" ) AND  ABS( man  OR  male*  OR  masculinit* ) AND  ABS( intervention  OR  training  OR  strateg*  OR  program* ) AND NOT  ABS( hiv  OR  aids  OR  immunodeficiency )
