# Supplementary material for: Mental Health Stigma Reduction Interventions Among Men: A Systematic Review
Source: Am J Mens Health. 2024 Nov 22;18(6):15579883241299353. doi: 10.1177/15579883241299353 (PMC11585030; doi:10.1177/15579883241299353)
Supplement: sj-pdf-2-jmh-10.1177_15579883241299353 – Supplemental material for Mental Health Stigma Reduction Interventions Among Men [file sj-pdf-2-jmh-10.1177_15579883241299353.pdf]

Code	Author & Year	Paper	Screening Questions for all types		Qualitative					Quantitative RCT					Quantititative Non-Randomised					Qunaitative Descriptive					Mixed Methods					Final Score	Strength	Reasons
			Are there clear qualitative and quantitative research questions (or objectives*), or a clear mixed methods question (or objective*)?	Do the collected data allow address the research question (objective)? E.g., consider whether the follow-up period is long enough for the outcome to occur (for longitudinal studies or study components).	1. Is the qualitative approach appropriate to answer the research question?	2. Are the qualitative data collection methods adequate to address the research question?	3. Are the findings adequetly derived from the data?	4. Is the interpretation of the results sufficiently substantiated by data?	5. Is there coherance between qualitative data sources, collection, analysis & interpretation?	Is randomisation appropriately performed?	Are the groups comparable at baseline?	Are there complete outcome data?	Are outcome assessors blinded by the intervention provided?	Did participants adhere to the assigned intervention?	Are participants representitative of the target population?	2. Are measurements appropriate (clear origin, or validity known, or standard instrument; and absence of contamination between groups when appropriate) regarding the exposure/intervention and outcomes?	Are there complete outcome data?	Are the confounders accounted for in the design and analysis?	During the study period, is the intervention administered (or exposure occurred) as intended?	1. Is the sampling strategy relevant to address the quantitative research question (quantitative aspect of the mixed methods question)?	2. Is the sample representative of the population understudy?	3. Are measurements appropriate (clear origin, or validity known, or standard instrument)?	Is the risk of nonresponse bias low?	Is the statistical analysis appropriate to answer the research question?	Is there an adequete rationale for using a mixed methods design to answer the research question?	Are the different components of the study effectively integrated to answer the research question?	Are the outputs of the integration of qualitative and quantitative components adequately interpreted?	Are divergences and inconsistencies between quantitative and qualitative results adequately addressed?	Do the different components of the study adhere to the quality criteria of each tradition of the methods involved?			
7	Morrow 2019	Intersectionality as an analytic Framework for understanding the Experiences of mental health stigma amoung racialized men	Yes	Yes	Yes	Yes	Can't tell	Yes	Yes																					80%	Moderate	1. Yes. 2. Yes. 3. Can't tell - does not report on data analysis method and does not use participant names/ages/focus group numbers not reported so can't tell where it is derived from. 4. Yes 5. Yes - clear link between al
1	Fung 2021a	Exploring mechanisms of mental illness stigma reduction in Asian Canadian Men	Yes	Yes						Can't tell	Can't tell	Yes	Can't tell	Can't Tell																20%	Weak	1. Generic statement "participants were randomly assigned" but does not state how. 2. does not report differences in participant baseline data once randomised, cannot determine if comparable or not. 4. Not reported if outcome assesors were blinded to intervention
4	Kohrt 2021	Collaboration with people with lived experience of mental illness to reduce stigma and improve primary care services	Yes	Yes						Yes	Yes	Yes	Yes	Can't Tell																80%	Moderate	1.Randomization well described. 2. Baseline charecteristics desribed, no significant differences. 3. Yes, 73-76% completed intervention and control. 4. States assessors are blinded in abstract. 5. Does not report on adherence/compliance to intervention
8	Nickerson 2019	Tell your Story. A Randomised controlled trial of an online intervention to reduce mental health stigma and increase help-seeking in refugee men with ptsd	Yes	Yes						Yes	Yes	Yes	Can't Tell	No																60%	Moderate	1. Yes computer randomisation clearly stated. 2. Yes, clearly stated. 3.Yes - 88.8% of data completed. (103 participants in total, 4 variables and 3 time points (total of 1236 possible responses [103x4x3].403 responses at basline, 329 responses at post, 366 responses at follow-up [1098 in total]. 1098 responses/1236 possible responses = 88.8% completion rate. 4. Does not report. 5. No. Average of 4.76 modules completed out of 11 (43% adherance rate)
13	Syzdek 2013	A pilot trial of gender-based motivational interviewing for hel-seeking and internalizing symptoms in men	Yes	Yes						Can't tell	Can't tell	Yes	Can't tell	Yes																40%	Weak	1. Does not report on randomisation process. 2. Does not report differences in baseline charecteristics, only key variables at basline. 3. High % of people report at 1 and 3months 4. Not reported. 5. Yes. Single session
16	Fung 2021b	Examining Different Strategies for Stigma Reduction and Mental	Yes	Yes						Can't tell	Can't tell	Yes	Can't tell	Can't tell																20%	Weak	1. Does not report on randomisation process. 2. Does not report differences in baseline charecteristics following randomisation. 3. Yes - 87% completion rate 4. Does not report 5. Does not report adherence to interventions
2	Hanisch 2017	Development and evaluation of digital game-based training for managers to promote employee mental health and reduce mental illness stigma at work	Yes	Yes											Yes	No	No	No	Yes											40%	Weak	1. 48 of 59 available sample participated. 2. No - developed own quiz, OMS-WA psychometric properties not validated. 3. No, 79% completion at follow-up . 4. Only accounted for age and education. DId not account for other demographics 5. Yes, appears people participated as intended (single session)
11	Sayer 2019	Does help seeking behabiour change over time following a workplace mental health intervention in the coal mining industy	Yes	Yes											No	Yes	Yes	Yes	Can't tell											60%	Moderate	1. No, only conducted with one company and two mines. 2. Yes - stigma included in "barriers to help-seeking" 3. Yes, 95% complete. 4. Yes, accounted for in analysis. 5. Does not report
12	Shimotzu 2014	Effectiveness of group CBT in reducing self stigma in japanese psychiatric patients	Yes	Yes											No	Yes	Yes	No	No											40%	Weak	1. No. Sampling from one clinic, population not described in depth. 2. Yes, clearly described. 3. Yes, 6% drop-out rate 4. No, background charecteristics or other treatements not accounted for in analysis. 5. Participants may have been exposed to other treatments during intervention and not accounted for.
14	Tynan 2018	Feasibility and acceptability of strategies to address mental health and mental ill-health in the australian coal mining industy	Yes	Yes											Yes	Yes	No	No	Yes											60%	Moderate	1. 8/10 mines invovled, stratification to ensure representative cross-section of industry. 2. Yes, clearly described. 3. No - average of site repsonse rates 75.7% (for WWMHP & control) 4. No - did not account for any confounders 5. Yes, single session & appears it was delivered as planned
15	Voorhees 2012	Pilot study of internet-based early intervention for combat-related mental distress	Yes	Yes											Yes	No	No	No	Yes											40%	Weak	1. Target participants well described. 2. Developed own scale to assess attitudes towards mental health and only selected some of these questions. 3. No - 72% completion rate across all timepoints (144 responses out of possible 200) 4. No did not account for any confounders 5. Yes appeared to be delivered as intended
10	Robinson 2014	Influencing public awareness to prevent male suicide	Yes	Yes																					No	No	No	No	No	0%	Weak	1. No - just states that the evaluation was complex. 2. No - reports the qual and quant data seperately without any attempt to integrate data. 3. No - does not integrate data in interpretation 4. No - does not report any differences between qual and quant data. 5. No - does not adequently report on the descriptive design element of this study
17	Woods 2020	Developing mental health awareness and help-seeking in prison.	Yes	Yes																					No	No	No	Yes	Yes	40%	Weak	1. No - does not provide a rationale for mixed methods study. 2. No - reports the qual and quant data seperateed without any attempt to integrate. 3. No - does not report on meta-interferace sufficently 4. Yes - discusses differences in findings between the two methods used.
